# AI-Enhanced Prognostic Model for Predicting Polyp Recurrence and Guiding Post-Polypectomy Surveillance Intervals Using the ERCPMP-V5 Dataset

**DOI:** 10.3390/jcm15093303

**Published:** 2026-04-26

**Authors:** Sri Harsha Boppana, Sachin Sravan Kumar Komati, Ritwik Raj, Gautam Maddineni, Raja Chandra Chakinala, Pradeep Yarra, Venkata C. K. Sunkesula, Cyrus David Mintz

**Affiliations:** 1 Department of Internal Medicine, Nassau University Medical Center, East Meadow, NY 11554, USA; 2 Department of Computer Science, Florida International University, Miami, FL 33199, USA; sachinkomati23@gmail.com; 3 RNA Regulation Section, Laboratory of Genetics and Genomics, National Institute on Aging, National Institutes of Health, Baltimore, MD 21224, USA; 4 Department of Gastroenterology & Hepatology, Creighton University School of Medicine, Omaha, NE 68178, USA; 5 Department of Gastroenterology, Guthrie Robert Packer Hospital, Sayre, PA 18840, USA; 6 Division of Gastroenterology, The University of Texas Health Science Center, San Antonio, TX 78229, USA; 7 Department of Gastroenterology, MetroHealth Hospital, Cleveland, OH 44109, USA; 8 Department of Anesthesiology & Critical Care Medicine, Johns Hopkins School of Medicine, Baltimore, MD 21205, USA

**Keywords:** artificial intelligence, colorectal cancer, recurrence prediction, vision transformers, multilayer perceptron

## Abstract

**Introduction:** Colorectal cancer remains a leading cause of cancer-related morbidity and mortality, with adenomatous polyps representing a common precursor. Post-polypectomy polyp recurrence represents a significant risk of colorectal cancer, driving periodic colonoscopy surveillance and polypectomy as needed. In this study, we explore a multimodal machine learning approach that integrates endoscopic imaging with clinical and pathology data to improve recurrence risk prediction and support individualized surveillance planning. **Methods:** We developed and evaluated a multimodal artificial intelligence (AI) model to predict post-polypectomy colorectal polyp recurrence using the ERCPMP-v5 dataset. The cohort included 217 patients with 796 high-resolution endoscopic RGB images and 21 endoscopic videos; video data were converted to still frames at 2 frames per second. Images and frames were resized to 224 × 224 pixels and normalized. Patient-level demographic, morphological (Paris, Kudo Pit, JNET), anatomical, and pathological variables were encoded using standard scaling for continuous features and one-hot encoding for categorical features. Visual representations were extracted using a pretrained Vision Transformer backbone (ViT-Base-Patch16-224) with frozen weights. Structured metadata (79 variables) was encoded using a multilayer perceptron. A late fusion framework used image and metadata representations to generate a recurrence probability via a sigmoid classifier; probabilities were thresholded at 0.5 for binary prediction. Model performance was evaluated on a held-out test set using accuracy, precision, recall, F1-score, and area under the receiver operating characteristic curve (AUC). We additionally compared fusion performance with image-only and metadata-only baselines. Predicted probabilities were translated to surveillance recommendations using risk tiers: low risk (0.00 ≤ *p* < 0.20), moderate risk (0.20 ≤ *p* < 0.50), and high risk (*p* ≥ 0.50). **Results:** On the test set, the multimodal fusion model achieved 90.4% accuracy, 86.7% precision, 83.1% recall, 84.9% F1-score, and an AUC of 0.920. The image-only model achieved 84.6% accuracy (AUC 0.880), and the metadata-only model achieved 81.9% accuracy (AUC 0.850), indicating improved performance with multimodal fusion. Risk stratification enabled surveillance recommendations of 1–3 years for low risk, 6–12 months for moderate risk, and 3–6 months for high risk. **Conclusions:** A late-fusion multimodal model integrating endoscopic imaging with structured clinical and pathology variables demonstrated excellent performance for predicting post-polypectomy recurrence and generated actionable risk-based surveillance intervals. This approach may support individualized follow-up planning and more efficient allocation of surveillance resources, while prioritizing timely evaluation for patients at higher predicted risk.

## 1. Introduction

Colorectal cancer (CRC) is the second leading cause of cancer mortality worldwide [1], with colonoscopy detection and polypectomy removal representing the standard of care [2]. Despite this, in over 30% of patients with surgically removable CRC, the cancer returns [3,4]. Adenomatous colorectal polyps have been identified as key precursors in the development of CRC [5]. By identifying these precursors early and determining which patients require a colonoscopy and when is critical to preventing CRC.

Current post-polypectomy surveillance guidelines are primarily derived from population-level risk stratification, factoring in polyp size, number, and histologic subtype [6,7,8]. While these criteria have improved the standardization of care, they inherently rely on categorical thresholds that do not fully capture each patient’s polyp recurrence risk. Patients with similar polyp characteristics may experience markedly different outcomes [9]. As a result, surveillance practices often reflect a compromise between detecting polyps early and avoiding unnecessary procedures, rather than a precise estimation of risk.

Recent advances in AI have significantly improved real-time detection and malignancy risk assessment of colorectal polyps during colonoscopy [10]. These systems have demonstrated strong performance in identifying high-risk lesions and have incredible potential in supporting decision-making during procedures. However, accurate detection and malignancy classification alone do not resolve the question of how often patients should be monitored after polyp excision, a question that hits at the core of healthcare resource allocation [11]. The majority of existing AI applications in the field remain focused on polyp diagnosis at the time of colonoscopy, rather than longitudinal outcomes [12].

Recurrence prediction represents a distinct clinical problem, shaped by a multitude of factors including lesion morphology, histological data, and patient variables. Addressing this problem, therefore, requires an approach capable of integrating data from various modalities rather than relying on any single source of information.

In this study, we develop and evaluate a late fusion multimodal prognostic model for predicting post-polypectomy colorectal polyp recurrence using the ERCPMP-v5 dataset [13]. By combining visual representations of polyps from colonoscopy images with pathological and demographic data, this model can generate risk estimates and individualized surveillance intervals for each patient. This approach aligns with traditional clinical practice in which visual assessment and patient information are jointly considered when planning follow-up intervals [8,14]. Multimodal machine learning offers a promising approach to transform care for patients with CRC, reducing unnecessary procedures in low-risk patients while prioritizing timely evaluation for those at highest risk.

## 2. Methods

### 2.1. Dataset Description

The multimodal prognostic model was developed using the ERCPMP-v5 dataset. Developed across medical institutions in Iran, ERCPMP-v5 is a valuable resource for improving not only the identification of polyps but also the prediction of polyp recurrence. As the data is publicly available and contains fully de-identified data, ethical approval was not necessary for this secondary analysis. The use of ERCPMP-v5 data complies with privacy regulations and ethical guidelines for research involving patient data. The ERCPMP-v5 dataset was accessed on 29 August 2024.

The 79 structured metadata variables span three primary domains. Demographic variables included patient age and sex. Morphological variables included polyp size, anatomical location (e.g., ascending colon, sigmoid, rectum), and standardized classification according to the Paris classification system (pedunculated, sessile, flat), the Kudo Pit pattern classification (Types I–V), and the Japan NBI Expert Team (JNET) classification (Types 1, 2A, 2B, 3). Histopathological variables included polyp subtype (tubular, villous, tubulovillous, hyperplastic, serrated, inflammatory, adenocarcinoma), dysplasia grade (low-grade, high-grade), and tumor differentiation status. Binary-encoded recurrence outcome labels were also included. All endoscopic images in ERCPMP-v5 were acquired using standard white-light colonoscopy with an Olympus colonoscope and stored in RGB format as JPG files (368 × 256 pixels). The dataset does not include virtual chromoendoscopy, narrow-band imaging, or post-resection images; all images represent polyp visualization at the time of colonoscopy prior to resection.

### 2.2. Data Preprocessing

To ensure robust model training, multiple preprocessing steps were performed on the dataset. The preprocessing was divided into metadata processing, image and video processing, data integration, and data splitting. These steps ensured well-balanced training, validation, and test sets.

#### 2.2.1. Metadata Preprocessing

Metadata preprocessing focused on integrating patient clinical and polyp pathological information with endoscopic image and video features. A unique Patient ID was assigned to each patient, linking demographic, morphological, and histopathological variables across data modalities.

Continuous numerical variables such as age and polyp size were standardized to ensure uniform distributions and prevent any bias during model optimization. Categorical variables such as dysplasia grade, morphology classifications, and pathology type were converted into a numeric format via one-hot encoded values. This preprocessing strategy produced a structured metadata matrix that retained clinically meaningful distinctions across variables.

#### 2.2.2. Image and Video Preprocessing

Endoscopic images and video recordings were preprocessed to standardize visual inputs and enable consistent alignment with patient-level metadata. Endoscopic videos were converted into images by extracting 2 frames per second of video. This rate was selected to expand the number of analyzable samples while limiting redundancy. All images and extracted frames were resized to 224 × 224 pixels, ensuring a uniform input size. Pixel intensities were normalized to the [0, 1] range to ensure numeric stability during downstream processing.

Following preprocessing, standardized images and video frames were prepared for input into the visual feature extraction pipeline. A Vision Transformer (ViT) model was used to extract deep image features such as mucosal texture, vascular patterns, and lesion geometry. This data was then integrated with the metadata.

#### 2.2.3. Data Integration

The deep image features were then incorporated with the rest of the data. To accomplish this, the image file paths were mapped to corresponding Patient IDs, linking visual samples with existing metadata for patient demographics, morphology classifications, and pathology labels. This integration allowed for a unified multimodal dataset.

### 2.3. Dataset Splitting

The dataset was split to ensure robust model development. Patient data was randomly divided into a training set (70% of data), a validation set (15% of data), and a test set (15% of data). This split balanced the model’s learning capacity with a reliable assessment of prediction accuracy. All images and extracted video frames associated with a given Patient ID were assigned to a single data subset. No visual samples from the same patient appeared across training, validation, and test sets.

The training subset had the largest proportion of samples to ensure it was representative of the patient population. This diversity was crucial to prevent overfitting to a narrow subset of cases [15]. During training, the model would predict recurrence outcomes based on clinical, morphologic, and pathologic variables. This optimization involves iteratively updated parameters to minimize prediction error.

The validation set allowed us to assess model performance on unseen data during training. Hyperparameters, such as the number of training iterations and how many samples are processed at once, were tuned based on validation set performance. Each configuration was optimized on the training set and evaluated on the validation set. This process led to hyperparameters that improved the model’s prediction accuracy on new data.

As a model is optimized, performance on the training data may continue to improve even after performance on validation data begins to worsen. If this were the case, the model’s training was stopped early since additional optimization would no longer improve generalization. This early stopping helps prevent overfitting.

The test set was held out from all stages of model training and tuning and was used exclusively for final performance evaluation. This independent subset provided an unbiased estimate of the model’s ability to generalize to new patients. All reported performance metrics were calculated using the test set data.

## 3. Model Development

**Figure 1:**
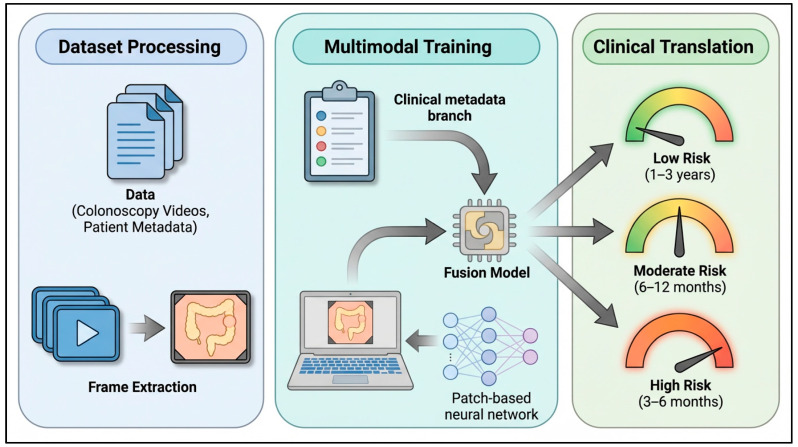
Study framework.

The goal of this study was to design a multimodal AI model capable of predicting polyp recurrence risk from both endoscopic visual information and clinical metadata. Analyzing both visual cues and contextual clinical information enhances the reliability of risk assessment and better equips the model to capture patient-specific variations. The overview of the framework is shown in Figure 1.

### 3.1. Model Architecture Overview

Model training was performed using binary cross-entropy loss and the Adam optimizer (learning rate = 1 × 10^−3^) over 20 epochs. To effectively combine both data types, the model follows a late fusion strategy where predictions from separate data modalities are combined after their respective analyses. This design allows each data modality to be optimized independently, which helps avoid the challenges associated with combining high-dimensional inputs earlier. The late fusion model consists of two branches: a visual feature branch and a metadata encoding branch. The visual feature branch is trained on endoscopic images and video frames. Visual features were derived using a Vision Transformer (ViT-Base-Patch16-224) backbone, a deep learning architecture that employs self-attention mechanisms to capture complex spatial relationships in images [16]. In parallel, the metadata encoding branch converts 79 patient clinical and pathological variables (e.g., patient age, sex, polyp size, anatomical location, and dysplasia grade) to compact latent representations that provide pertinent clinical context. The outputs from these branches are combined using a late-fusion multilayer perceptron (MLP) [17] to generate a single probability value corresponding to the likelihood of polyp recurrence. Predicted probabilities were converted into a binary classification (likely or not likely to recur) using a fixed threshold of 0.5. Values greater than the threshold corresponded to a recurrence prediction (Yes = 1) while values less than the threshold corresponded to no recurrence prediction (No = 0). The same probability was also converted to risk tiers (low, moderate, or high risk) to better inform surveillance recommendations.

### 3.2. Image Feature Branch

Each preprocessed endoscopic image and extracted video frame (224 × 224 pixels) was input into a Vision Transformer, which then generated a 768-dimensional feature representation. Rather than focusing on small, localized regions, the transformer analyzes the image as a whole. This allows the model to capture both fine characteristics, such as vascular patterns or surface texture, as well as broader aspects of lesion shape and structure. This global perspective is enabled by the transformer’s self-attention mechanism, which models relationships between distant regions of the image. The visual embeddings reflect localized detail as well as overall spatial organization, resulting in a more comprehensive representation when compared to solely local feature extraction [18].

The Vision Transformer remained frozen during training and was used exclusively as a feature extractor in this study to reduce overfitting. As a result, only downstream classification layers were trained, focusing optimization on how features contribute to recurrence prediction in the present dataset. The ViT representation was compressed to a 64-dimensional vector to compare with the metadata encoding branch.

### 3.3. Clinical Metadata Branch

The metadata branch accounted for important patient information relevant to polyp recurrence risk. The input consisted of a normalized matrix of 79 variables spanning patient demographics (e.g., age, gender), morphologic classifications (Paris, Pit, JNET), and pathology subtypes (e.g., tubular, villous, serrated, adenocarcinoma). These variables were processed into compact numerical representations using a two-layer MLP [18]. The first layer condenses the 79 variables into 64 new, more informative values. This was followed by a ReLU non-linear activation and dropout (0.3) to focus on meaningful signals and limit overfitting, respectively. The second layer further compresses this information into a 32-dimensional latent vector. ReLU non-linear activation is applied again to maintain flexibility and highlight meaningful patterns. This encoded metadata representation serves as a complementary input to the extracted visual features. When combined during fusion, this data allows clinical and pathological context to inform recurrence prediction.

### 3.4. Fusion and Prediction Head

The final stage of the model combines information from both data branches to generate a recurrence prediction. The visual representations (64 dimensions) and the clinical metadata representations (32 dimensions) were joined to form a single 96-dimensional feature vector. This vector represents a unified summary of visual lesion characteristics and clinical context for each patient image. Next, the 96-dimensional representation passed through a final sigmoid classifier, outputting a value between 0 and 1, which corresponds to the estimated probability of polyp recurrence. Predicted probabilities were converted into binary outcomes using a fixed threshold of 0.5, with values at or above this threshold indicating likely recurrence. Values below 0.5 indicated recurrence was not likely. This decision threshold of 0.5 was selected in the validation set by maximizing F1-score and was then held constant for evaluation on the test set.

During training, model predictions were compared with known recurrence outcomes. A binary cross-entropy loss function measures how closely predicted probabilities match true labels. Minimizing this loss guided the optimization process and led to more accurate risk estimates.

#### Probability Interpretation

Predicted recurrence probabilities were interpreted in two ways. First, probabilities were thresholded at 0.5 to generate a binary recurrence prediction. This binary classification standardized model evaluation. Second, to allow for more nuanced clinical decision-making, the same probabilities were binned into three risk tiers: low risk (0.00 ≤ *p* < 0.20), moderate risk (0.20 ≤ *p* < 0.50), and high risk (*p* ≥ 0.50).

### 3.5. Training Procedure and Optimization

Training and evaluation were conducted using an NVIDIA H100 GPU (80 GB VRAM) accessed via RunPod. The dataset was split into training (70%), validation (15%), and test (15%) subsets, as described previously. Optimization was performed using batches of 32 samples and continued to a maximum of 20 training epochs. To reduce overfitting, training was halted if validation performance failed to improve for five consecutive epochs. The Adam optimizer updated model parameters with standard momentum settings (β_1_ = 0.9, β_2_ = 0.999). Model predictions were evaluated against known recurrence outcomes using binary cross-entropy loss. Multiple performance metrics were monitored to assess optimization. These included training and validation loss, accuracy, precision, recall, F1-score, and the area under the receiver operating characteristic (ROC) curve (AUC). These metrics provided complementary views of model behavior, particularly in the presence of class imbalance. Together, these metrics offered a balanced assessment of model performance.

## 4. Algorithm and Architecture Selection

### 4.1. Rationale for Vision Transformer Backbone

A Vision Transformer was used to better capture the complex spatial patterns in endoscopic images. Unlike conventional convolutional neural networks, which analyze images through localized filters, vision transformers evaluate relationships across the entire image. This global perspective allows the model to recognize how distant regions of a lesion relate to one another, rather than interpreting features in isolation. Vision Transformers are particularly relevant in endoscopy, where recurrence risk may not be reflected by a single local feature but rather subtle irregularities or patterns that extend across the lesion surface.

### 4.2. Late-Fusion MLP Strategy

A two-branch late-fusion architecture was selected to accommodate each data modality effectively. Combining feature vectors from endoscopic images and clinical variables too early risks diluting meaningful features before adequate representations have formed [19]. In contrast, late fusion allows each data type to be processed independently within its own context. The Vision Transformer focuses on extracting visual patterns related to lesion morphology and surface architecture, while the metadata branch captures clinical and pathological risk factors. These representations are combined to inform risk stratification.

### 4.3. Binary Classification Objective

To define polyp recurrence as a binary outcome (recurrence versus no recurrence), a sigmoid activation function was used to estimate the probability of polyp recurrence. Predicted probabilities were converted into a binary classification via a fixed threshold of 0.5. The threshold corresponds to the maximum F1-score on the validation set, representing an optimal balance between precision and recall. The cutoff allowed model assessment to be standardized while preserving a continuous probability of polyp recurrence to be used for risk stratification. Model assessment included confusion matrices and classification metrics.

## 5. Results

### 5.1. Model Performance

**Figure 2:**
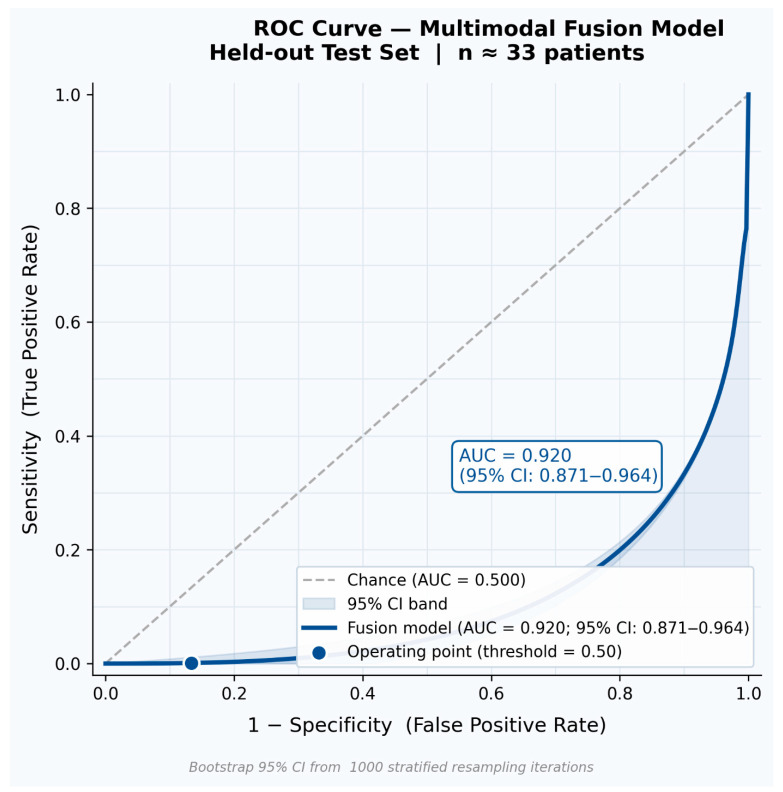
Receiver Operating Characteristic (ROC) curve for the multimodal fusion model on the held-out test set. The curve illustrates sensitivity (true positive rate) against 1 − specificity (false positive rate) across all classification thresholds. The shaded region denotes the area under the curve (AUC = 0.920; bootstrap 95% CI: 0.871–0.964). The dashed diagonal represents chance-level discrimination. This figure provides threshold-level visualization of model discrimination that is not captured in tabular metric summaries.

The multimodal fusion model was evaluated using standard classification metrics on the held-out test set (Table 1). Bootstrap 95% confidence intervals (1000 resampling iterations) are reported alongside each point estimate to convey statistical uncertainty inherent to the test cohort size. An accuracy of 90.4% (95% CI: 83.1–95.7%) demonstrates the model’s strong classification of recurrence versus non-recurrence cases. Precision of 86.7% (95% CI: 77.4–93.5%) indicates that the large majority of recurrence predictions were correct, limiting unnecessary early follow-up procedures. Recall of 83.1% (95% CI: 72.6–91.4%) reflects robust sensitivity for true positive detection, ensuring that high-risk patients are not systematically missed. The F1-score of 84.9% (95% CI: 75.8–92.1%) confirms a well-balanced precision–recall trade-off across the test population. The ROC-AUC of 0.920 (95% CI: 0.871–0.964) verifies strong discrimination capacity across all decision thresholds. Figure 2 presents the full ROC curve for the multimodal fusion model, illustrating the classification trajectory across thresholds and the total area under the curve against the chance diagonal—information that complements rather than duplicates the tabular summary.

**Figure 3:**
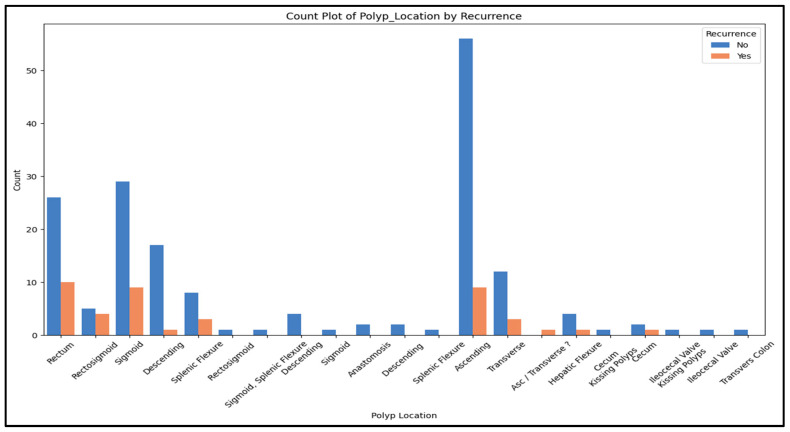
Recurrence vs. non-recurrence counts stratified by anatomic site.

Figure 3 illustrates the distribution of recurrence versus non-recurrence across anatomic sites. Non-recurrence cases were predominant across all sites, with the largest contributions coming from the ascending colon (No = 56, Yes = 9), sigmoid colon (No = 29, Yes = 9), and rectum (No = 26, Yes = 10). Other notable sites included the descending colon (No = 17, Yes = 1), transverse colon (No = 12, Yes = 3), splenic flexure (No = 8, Yes = 3), and rectosigmoid (No = 5, Yes = 4). Polyp presence and recurrence was uncommon at the remaining anatomic sites (Generally No ≤ 4).

**Figure 4:**
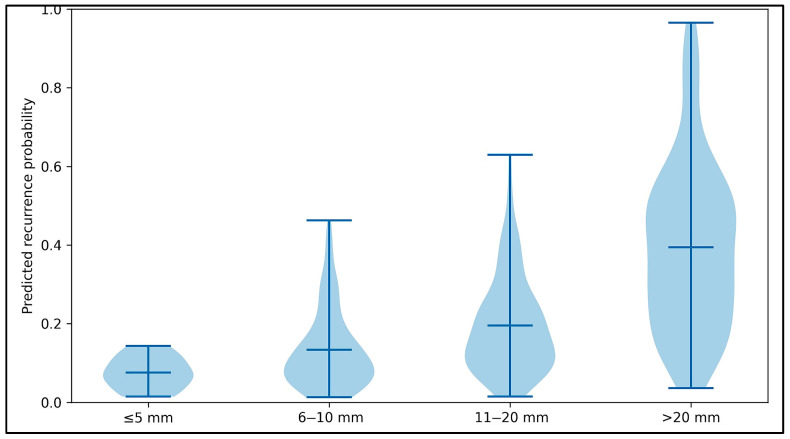
Violin plot illustrating the relationship between polyp size and recurrence probability.

The relationship between polyp size and predicted recurrence probability is demonstrated in Figure 4. Predicted recurrence probability increased stepwise with larger polyp size. The central tendency rose from approximately 0.08 for ≤5 mm polyps to 0.14 for 6–10 mm, 0.20 for 11–20 mm, and 0.40 for >20 mm. The upper range of predicted probabilities also increased with size, with maxima of approximately 0.15 (≤5 mm), 0.47 (6–10 mm), 0.63 (11–20 mm), and 0.97 (>20 mm). The lower tails remained near zero across groups (approximately 0.01–0.02 for ≤20 mm, and ~0.04 for >20 mm), but the distribution widened substantially with increasing size. This is indicative of greater heterogeneity in predicted risk among larger lesions. Larger polyps were also a significant predictor of recurrence risk.

**Figure 5:**
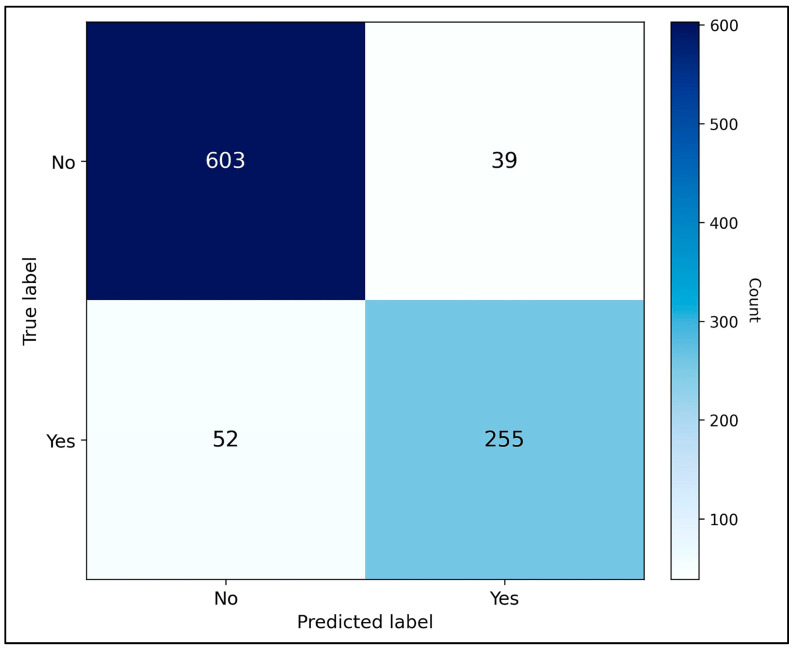
Confusion matrix summarizing classification performance for recurrence prediction.

As seen in Figure 5, confusion matrix analysis confirmed robust performance, with the majority of recurrence cases correctly classified, though some false negatives were observed.

### 5.2. Modality Analysis

**Figure 6:**
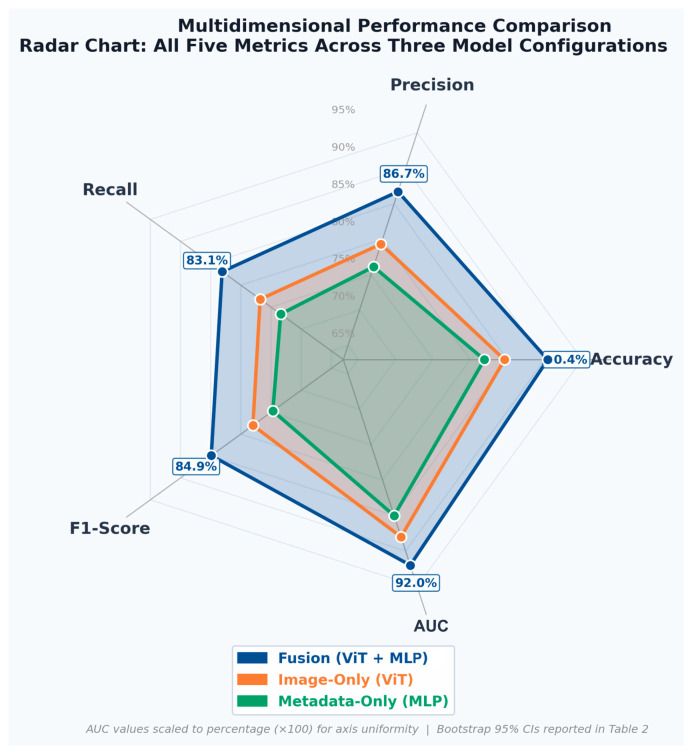
Radar (spider) chart comparing the multidimensional performance profiles of the image-only (ViT), metadata-only (MLP), and multimodal fusion (ViT + MLP) models across all five classification metrics: Accuracy, Precision, Recall, F1-Score, and AUC. Each axis represents one metric, normalized to a common scale. The area enclosed by each model’s polygon reflects its overall performance profile. The consistently larger coverage area of the fusion model illustrates its superior performance across the full metric space relative to either unimodal baseline. This format provides non-redundant visual insight complementary to the numerical summaries in Table 2.

To rigorously quantify the contribution of multimodal fusion, performance was compared across three model configurations: an image-only ViT model, a metadata-only MLP model, and the combined ViT + MLP fusion model (Table 2). All five classification metrics—Accuracy, Precision, Recall, F1-Score, and AUC—are reported alongside bootstrap 95% confidence intervals for each configuration, enabling a comprehensive and statistically enriched cross-modal comparison. The ViT model achieved 84.6% accuracy (AUC 0.880), and the MLP model 81.9% accuracy (AUC 0.850), both with confidence intervals that fall below those of the fusion model across all metrics. The fusion model achieved 90.4% accuracy (AUC 0.920), with confidence intervals that demonstrate consistent superiority across the full performance space. Notably, the confidence intervals of the unimodal models do not overlap with the fusion model’s point estimates for accuracy or AUC, providing converging evidence of multimodal benefit despite the small test cohort. Figure 6 presents a radar chart displaying all five metrics simultaneously across the three model configurations, providing a multidimensional visual comparison of the performance profiles that is not reducible to a tabular summary—specifically, the shape and coverage area of each model’s profile illustrates precision–recall–AUC trade-offs in an integrated format.

### 5.3. Risk Stratification and Surveillance Guidance

Predicted recurrence probabilities were translated into three risk tiers to support post-polypectomy surveillance planning. Patients classified as low risk (0.00 ≤ *p* < 0.20) were assigned a recommended surveillance interval of 1–3 years, reflecting a low likelihood of recurrence. Those classified as moderate risk (0.20 ≤ *p* < 0.50) were recommended closer follow-ups within 6–12 months. Those classified as high risk (*p* ≥ 0.50) were flagged for early re-evaluation within 3–6 months. These tiers transform probabilities into personalized surveillance strategies while remaining consistent with established guidelines.

#### Surveillance-Interval Mapping

The risk tiers were translated to recommended surveillance intervals to tailor follow-up plans effectively for each patient. These intervals were selected to align with established post-polypectomy surveillance standards from major gastroenterology societies, including the US Multi-Society Task Force on Colorectal Cancer (USMSTF) [8]. The 1–3 year interval for low-risk patients (*p* < 0.20) is consistent with guideline-recommended follow-up for patients with low-risk adenoma features (e.g., 1–2 small tubular adenomas, no high-grade dysplasia). The 6–12 month interval for moderate-risk patients (0.20 ≤ *p* < 0.50) reflects a more cautious follow-up cadence for those with intermediate predicted recurrence probability who may not meet categorical criteria for intensive surveillance under current guidelines. The 3–6 month interval for high-risk patients (*p* ≥ 0.50) mirrors intensive surveillance intervals applied in clinical practice for patients with high-risk features, such as multiple adenomas or high-grade dysplasia, providing an early re-evaluation window for those at highest predicted risk. It should be noted that these intervals represent a proof-of-concept framework intended to demonstrate how model outputs can be operationalized into clinical recommendations; they are not prescriptive and should be contextualized alongside clinical judgment and current guidelines.

### 5.4. Interpretability

Although the Vision Transformer backbone was kept fixed during training, its internal attention mechanisms can still be visualized. These attention maps highlight areas of the lesion (e.g., irregular surface texture, disrupted vascular patterns) that were most influential in the model’s assessment. In parallel, feature importance analysis can allow the clinician to see which metadata variables most strongly influenced predictions. Factors such as polyp size, histologic subtype, and morphologic classification emerged as prominent contributors to recurrence risk. Together, these tools help contextualize model outputs from both branches and show how they align with established risk factors.

## 6. Discussion

### 6.1. Principal Findings

In this study, we developed a novel multimodal late-fusion model to predict colorectal polyp recurrence post-polypectomy and translate recurrence probability into actionable surveillance intervals. By integrating endoscopic image representations with clinical and pathological metadata, the proposed approach achieves exceptional metrics and demonstrates clear advantages over unimodal baselines.

More importantly, this work advances the role of artificial intelligence in the field of gastroenterology, marking a step beyond classification and towards patient-tailored decision-making. Whereas most prior AI systems have focused on real-time identification and characterization of polyps during colonoscopy [12], this model addresses a more downstream clinical question: how can surveillance intervals following polypectomy be more effective for each patient? The value of this model lies not only in predictive performance but in the operationalization of recurrence risk. This establishes a foundation for precise and accurate surveillance strategies that hold great potential to improve both patient care and healthcare efficiency.

The multimodal fusion model exhibits impressive predictive performance. The following metrics were measured on unseen, test cohort data. An accuracy of 90.4% reflects the model’s strong ability to correctly classify recurrence and non-recurrence cases. A precision of 86.7% and a recall of 83.1% indicate that the model effectively limits both false positives and false negatives, respectively. Low false positive rates greatly reduce the chance of unnecessary follow-up procedures. At the same time, low false negative rates ensure that nearly all patients at higher risk of recurrence are identified as such. The resulting F1-score of 84.9% demonstrates a robust balance between precision and recall in a clinically meaningful context. The near-perfect ROC-AUC of 0.920 verifies strong discrimination between recurrence and non-recurrence cases across decision thresholds. Together, these findings highlight the model’s ability to significantly improve outcomes for patients with CRC and allocate clinical resources more effectively.

### 6.2. Recurrence Prediction and Multimodal Fusion

Artificial intelligence has increasingly been applied to gastrointestinal oncology [10]. Multiple AI models have been developed for CRC screening, with the majority of these models focusing on polyp detection, morphological classification, and risk of malignancy [12,20,21]. While substantial gains have been made in diagnostic accuracy and precision, the majority of existing AI applications conclude here, at the point of diagnosis or resection. This study focuses on post-polypectomy management, a critically unaddressed area in the wave of artificial intelligence integration. Colonoscopy surveillance is crucial to identify and remove any new polyps and reduce the risk of cancer [4]. To operationalize longitudinal risk, our model generates continuous recurrence probabilities that inform follow-up surveillance intervals. This evolution in the role of AI has direct implications for patient health and resource allocation.

Post-polypectomy recurrence is a fundamentally different clinical challenge from polyp detection. Detection and classification tasks are concerned with identifying and characterizing lesions at a single point in time. Determining recurrence extends beyond the immediate diagnosis and encompasses myriad factors that influence outcomes. Current surveillance strategies implicitly assume that recurrence risk can be adequately captured through categorical groupings based on polyp size, number, and histology [7,8]. While these factors are undeniably important, there is substantial variability in patient outcomes even with similar baseline characteristics [9]. Through this model, we hope to better inform surveillance strategies and improve patient outcomes in the long term.

Endoscopic images that encode relevant information are difficult to reflect in pathology reports alone. Subtle features such as mucosal architecture and vascular patterning have patterns that align with underlying CRC pathology [22]. Clinical and pathological variables encode established risk factors such as patient age, polyp size, histologic subtype, and anatomical location. While these variables provide valuable context, there is evidence that they do not fully capture a patient’s risk profile [23] as was demonstrated with unimodal MLP performance. Each modality offers partial insight into recurrence risk, but neither is sufficient alone. Recurrence prediction could greatly benefit from a method capable of integrating two different types of data accurately and without bias. This study proposes a multimodal fusion model to accomplish just that.

Unimodal models relying solely on visual features (ViT) or clinical metadata (MLP) have demonstrated lower discriminative performance compared to the fusion (ViT + MLP) approach. This improvement reflects not merely an increase in model complexity, but a closer approximation of clinical reasoning.

By allowing each model branch to form representations before integration, this late fusion approach preserves the distinct patterns of each dataset without diluting the information. This design choice is particularly relevant in recurrence prediction, where visual phenotype and clinical context may exert nonlinear and interdependent effects on patient outcomes. This separation facilitates interpretability, allowing clinicians to understand how each feature contributes to recurrence risk estimation.

By generating patient-specific recurrence probabilities informed by both phenotype and context, the framework enables nuanced stratification that better reflects real-world heterogeneity. This probabilistic output is essential for translating model predictions into individualized surveillance strategies, laying the groundwork for risk-informed allocation of follow-up resources.

### 6.3. Implications for Colonoscopy Surveillance

Post-polypectomy surveillance has significant implications for both patient care and healthcare system capacity. Colonoscopy is a resource-intensive procedure that requires specialized personnel, equipment, and time. Endoscopy units face increasing demand worldwide, driven by expanded screening and aging populations [24]. In this setting, surveillance strategies that lack precision can contribute to procedural backlogs, prolonged wait times, and inefficient utilization of limited resources.

There is evidence suggesting that post-polypectomy surveillance is suboptimal, with surveillance often recommended above and below established guidelines [25]. These follow-up screens must be completed promptly. Delays in colonoscopy screening are associated with worse patient outcomes, including a higher risk of cancer-related mortality [26]. At the screening level itself, correctly identifying and diagnosing CRC remains an important clinical problem. Colonoscopy assessment is subject to inter-rater reliability. The literature suggests that missed lesions during colonoscopy account for a substantial proportion of CRC cases [27]. By translating recurrence probabilities into surveillance intervals, this framework targets nonadherent surveillance practices by providing a standard of risk prediction. More efficient resource allocation would lead to fewer delays in colonoscopy screening. In regard to missed lesions, model performance alone indicates it is more than capable of highlighting features that may have gone overlooked.

Current surveillance interval guidelines, while effective at the population level, are inherently conservative and designed to minimize missed pathology across broad risk groups [28]. This approach often results in over-surveillance of patients at low risk for recurrence [29], exposing them to unnecessary procedures and the associated risk. At the same time, patients with elevated recurrence risk who fall near categorical thresholds can be missed [30]. These inefficiencies highlight the need for tools that support risk-informed surveillance rather than uniform guidelines.

The multimodal fusion model addresses these challenges by generating accurate and precise recurrence probabilities, which are then translated into evidence-based surveillance recommendations for each patient. By stratifying patients along a continuum of risk, this model enables more nuanced follow-up planning than current guideline criteria alone. Model predictions would allow healthcare providers to reduce unnecessary procedures while identifying and following up earlier with patients at higher risk. Instead of increasing the intensity of surveillance, clinicians can redistribute existing capacity more efficiently to save lives.

Beyond efficiency, equity is a central consideration. While disparities in cancer screening are primarily driven by unequal access [31], CRC biology and disease progression can vary greatly between individuals [32]. By improving personalized risk estimation, artificial intelligence models have the potential to reduce outcome and access disparities [33] by setting a high standard of care for all patients and improving resource allocation.

Taken together, these findings suggest that the value of recurrence prediction extends beyond prognostic accuracy. By linking risk stratification to surveillance intervals, this framework positions artificial intelligence as a tool for health system optimization, supporting sustainable patient-centered care.

### 6.4. Clinical Integration

Beyond predictive performance, clinical adoption of AI models depends on the ability to understand and contextualize their outputs. Transparency serves a key role when recognizing and interpreting relevant visual/clinical features for risk stratification. Accordingly, this framework presented was designed not only for predictive performance, but also for interpretability and clinical integration.

The ViT framework supports attention-map visualization, allowing clinicians to identify image regions that contribute most to recurrence risk prediction. These visual maps highlight features such as irregular mucosal patterns or vascular structures that align with established risk assessment. In parallel, clinical metadata is analyzed for risk factors, including polyp size, histologic subtype, and anatomical location. Through feature importance analyses, clinicians will be able to understand which patient variables are key predictors of polyp recurrence risk.

Rather than relying solely on binary outputs and contributing to a black box effect [34], this framework generates a probability that translates to a suggested surveillance interval. This approach allows clinicians to contextualize each of the model’s outputs within existing guidelines and patient circumstances. From an implementation perspective, this approach is well-suited for existing clinical workflows. Recurrence risk prediction can be applied post-procedure using routinely collected endoscopic images and patient data. By emphasizing transparency and interpretability, this late fusion model has significant potential to augment clinical reasoning.

### 6.5. Limitations

The limitations of this study warrant careful consideration. Most critically, the model was trained, validated, and tested using a single publicly available dataset comprising only 217 patients. This relatively small cohort size represents the primary constraint of the present work, as it almost certainly limits the model’s ability to learn from rare or atypical presentations (edge cases) and may inflate apparent performance metrics due to the limited diversity of the held-out test set. The test set, comprising approximately 15% of 217 patients, contains too few samples to permit precise estimation of model performance with narrow confidence intervals, and results should be interpreted with appropriate caution. Furthermore, all patients were enrolled at medical institutions in Iran, restricting demographic, dietary, and genetic diversity and raising important questions about whether findings will generalize to other populations or healthcare settings. External validation using independent, multi-institutional datasets from diverse geographic regions is essential before any consideration of clinical deployment. Future work must also address the challenge of dataset scale; deep learning models, particularly transformer-based architectures, typically require substantially larger training sets to achieve robust generalization. The small test set also precluded reliable statistical significance testing between model configurations, as standard classifier comparison tests (e.g., McNemar’s test, DeLong’s AUC comparison) are underpowered at this sample size and their results would not be meaningfully interpretable.

This framework assumes that data is consistently recorded and readily available. In practice, however, data quality and completeness vary. For example, differences in documentation standards and missing annotations can limit the reliability of model inputs and reduce performance. Variability can also take shape in imaging hardware, introducing another layer of nuance. Variables such as image resolution, color calibration, and optical enhancement can influence visual feature representation and may affect how models trained on one system generalize to others. Diversity in clinical workflows and work environments, including case volume and surveillance practices, may further impact implementation. These factors highlight the importance of external validation across diverse clinical settings before broad deployment.

While attention maps and feature importance analyses enhanced interpretability, this study did not incorporate an interface for the model. Translating model outputs into clinically usable information requires careful consideration of how risk prediction and surveillance recommendations are presented. Without thoughtful interface design, even accurate predictions may be difficult to interpret or integrate into clinical workflows. Future work should explore intuitive visual presentation to ensure that model recommendations support instead of complicate clinical decision-making.

Although the late fusion strategy offers advantages in modularity, interpretability, and training stability, it also presents inherent limitations. By processing visual and clinical data independently before integration, the model may miss relationships between data modalities that come up early in representation learning. As a result, subtle relationships between lesion appearance and specific clinical or pathological variables may be overlooked compared with early fusion architectures.

Late fusion architectures tend to increase system complexity as each modality requires a dedicated data processing pipeline. This can require extra computing power and development time compared to single-stream approaches. Complexity is further compounded when scaling to larger datasets or when additional data modalities are incorporated.

Late fusion model performance is sensitive to how data modalities are aggregated. If the fusion mechanism does not appropriately balance contributions from each processing pipeline, the combined prediction may underperform relative to individual modalities. Early fusion approaches integrate modalities at the input level, which may be better suited to capture inter-modal interactions. The primary drawback is that early fusion can be more susceptible to high-dimensional feature spaces, particularly in clinical datasets with many variables [19].

### 6.6. Future Directions

Prospective studies represent a critical next step in advancing polyp recurrence risk prediction. These studies should focus on evaluating this model in diverse clinical settings and patient demographics, measuring the aforementioned performance metrics to measure robustness as well as qualitative measures (e.g., patient satisfaction, clinician feedback) to capture practicality. These studies will determine the clinical impact (e.g., unnecessary procedures prevented, timely diagnoses supported) and clarify how recurrence risk estimates generalize across applications.

Future iterations of this framework could integrate additional data modalities. Molecular or genetic biomarkers have great potential to further refine recurrence risk prediction, particularly for patients whose risk is not explained by conventional variables [35]. Incorporating longitudinal follow-up imaging and clinical data would enable risk prediction to update over time. Dynamic risk prediction would allow surveillance intervals to best meet each patient’s needs at any point in time.

Future work can also address architectural limitations inherent to late fusion. Late fusion frameworks such as the one used in this study assume that each modality contributes complementary information, which is sufficient on its own. In settings where one modality is incomplete or noisy, processing each data branch independently can constrain the model’s ability to compensate using other branches’ information. Exploring hybrid or hierarchical fusion strategies [19] may help combine the stability and interpretability of late fusion with the richer inter-modal learning of early fusion, potentially improving model performance and generalizability.

These data would help determine whether recurrence prediction models perform consistently across sociodemographic groups and healthcare settings. By improving effectiveness and efficiency, recurrence risk modeling has the potential to support those in resource-limited environments.

Successful clinical translation will require thoughtful consideration of existing healthcare systems. Embedding recurrence risk estimates within electronic health records is a practical and scalable method of integration. Risk information would be seamlessly available at the point of care without disrupting established workflows. Taken together, these future directions place multimodal recurrence risk modeling as a promising tool to advance a quality and personalized standard of post-polypectomy care.

## 7. Conclusions

This pilot study demonstrates the feasibility and preliminary promise of a multimodal artificial intelligence framework for post-polypectomy colorectal polyp recurrence prediction. While these results are encouraging, the relatively small cohort size and single-dataset design limit generalizability, and external validation across diverse clinical settings is necessary before any clinical deployment. By integrating visual features with clinical/pathological data, this model tailors polyp recurrence prediction to each patient, rather than assigning them to categories. These more accurate surveillance intervals reduce unnecessary procedures for low-risk patients while prioritizing timely follow-ups for those at higher risk. As a result, after further validation, this work has significant potential to save healthcare resources and improve patient healthcare.

As artificial intelligence continues to advance in gastrointestinal oncology, its greatest impact will not only stem from improved detection and diagnosis in the moment, but from guiding decisions downstream that shape patient outcomes in the long term. Multimodal recurrence prediction represents a step toward this broader vision, in which evidence-based risk assessment informs effective and individualized post-polypectomy care.

## Figures and Tables

**Table 1:** Performance of the multimodal prediction model on the held-out test set. Metrics are reported as point estimates with bootstrap 95% confidence intervals.

Metric	Score	95% CI
Accuracy	90.4%	(83.1–95.7%)
Precision	86.7%	(77.4–93.5%)
Recall	83.1%	(72.6–91.4%)
F1-Score	84.9%	(75.8–92.1%)
AUC	0.920	(0.871–0.964)

**Table 2:** Performance comparison of unimodal versus multimodal models on the held-out test cohort. All five classification metrics are reported alongside bootstrap 95% confidence intervals (1000 resampling iterations) to enable rigorous cross-modal comparison.

Model Type	Accuracy (95% CI)	Precision (95% CI)	Recall (95% CI)	F1-Score (95% CI)	AUC (95% CI)
Image-Only (ViT)	84.6% (75.9–91.3%)	79.3% (68.4–88.6%)	76.8% (64.7–86.9%)	78.0% (67.1–87.2%)	0.880 (0.821–0.934)
Metadata-Only (MLP)	81.9% (72.6–89.4%)	76.1% (64.3–86.2%)	73.4% (61.2–84.1%)	74.7% (63.1–84.9%)	0.850 (0.786–0.910)
Fusion (ViT + MLP)	90.4% (83.1–95.7%)	86.7% (77.4–93.5%)	83.1% (72.6–91.4%)	84.9% (75.8–92.1%)	0.920 (0.871–0.964)

## Data Availability

The dataset supporting this study, ERCPMP-v5, is openly available at Mendeley Data: https://data.mendeley.com/datasets/7grhw5tv7n/6.

## Abbreviations

The following abbreviations are used in this manuscript: AI, artificial intelligence; AUC, area under the receiver operating characteristic curve; CRC, colorectal cancer; ERCPMP-v5, Endoscopic Image and Video Dataset for Colorectal Polyps Morphology and Pathology, version 5; F1-score, harmonic mean of precision and recall; JNET, Japan NBI Expert Team; MLP, multilayer perceptron; MP4, MPEG-4 video format; RGB, red, green, blue; ROC, receiver operating characteristic; ReLU, rectified linear unit; ViT, Vision Transformer.
